# Acute Stress Enhances Epigenetic Modifications But Does Not Affect the Constitutive Binding of pCREB to Immediate-Early Gene Promoters in the Rat Hippocampus

**DOI:** 10.3389/fnmol.2017.00416

**Published:** 2017-12-19

**Authors:** Sylvia D. Carter, Karen R. Mifsud, Johannes M. H. M. Reul

**Affiliations:** Neuro-Epigenetics Research Group, Bristol Medical School, University of Bristol, Bristol, United Kingdom

**Keywords:** stress, hippocampus, immediate-early gene, CREB, histone phospho-acetylation

## Abstract

The immediate early genes (IEGs) *c-Fos* and *Egr-1* are rapidly and transiently induced in sparse neurons within the hippocampus after exposure to an acute stressor. The induction of these genes is a critical part of the molecular mechanisms underlying successful behavioral adaptation to stress. Our previous work has shown that transcriptional activation of *c-Fos* and *Egr-1* in the hippocampus requires formation of a dual histone mark within their promoter regions, the phosphorylation of serine 10 and acetylation of lysine 9/14 of histone H3. In the present study, using chromatin immuno-precipitation (ChIP), we found that an increase in the formation of H3K9ac-S10p occurs within the *c-Fos* and *Egr-1* promoters after FS stress *in vivo* and that these histone modifications were located to promoter regions containing cAMP Responsive Elements (CREs), but not in neighboring regions containing only Serum Responsive Elements (SREs). Surprisingly, however, subsequent ChIP analyses showed no changes in the binding of pCREB or CREB-binding protein (CBP) to the CREs after FS. In fact, pCREB binding to the *c-Fos* and *Egr-1* promoters was already highly enriched under baseline conditions and did not increase further after stress. We suggest that constitutive pCREB binding may keep *c-Fos* and *Egr-1* in a poised state for activation. Possibly, the formation of H3K9ac-S10p in the vicinity of CRE sites may participate in unblocking transcriptional elongation through recruitment of additional epigenetic factors.

## Introduction

Immediate early genes (IEGs) such as FBJ osteosarcoma oncogene (*c-Fos*) and Early growth response 1 (*Egr-1*) are induced rapidly in response to a challenge. This rapid response is the result of a complex cascade of molecular events including intracellular signaling, chromatin modifications and transcription factor binding in the promoter regions of the genes (Gutierrez-Mecinas et al., 2011; O’Donnell et al., 2012; Reul, 2014). Transcriptional control of the IEGs *c-Fos* and *Egr-1* is traditionally thought to occur predominantly by the activation of the ERK MAPK signaling cascade, which then serves to phosphorylate and activate transcription factors such as cAMP response element binding protein (CREB) and ETS-domain containing protein 1 (Elk-1) (Treisman, 1985; Whitmarsh et al., 1995; Sgambato et al., 1998; Davis et al., 2000). Within the *c-Fos* and *Egr-1* promoter regions, CREB binds to cAMP responsive elements (CREs) whereas a complex of serum response factor (SRF) and pElk-1 (phosphorylated ETS domain-containing protein) binds to serum response elements (SREs) (Changelian et al., 1989; Defranco et al., 1993; Robertson et al., 1995). A series of experiments in transgenic mice expressing a lacZ reporter gene under the control of mutated forms of the Fos promoter showed that both the CRE and the SRE are important for regulation of *c-Fos* expression (Robertson et al., 1995).

CREB is a constitutively expressed protein, which is activated rapidly by phosphorylation at serine 133 (Ser133). Although some studies have found that CREB is constitutively bound to its recognition sites within the genome, the transactivation activity of CREB at those sites [i.e., recruitment of co-factors such as CREB binding protein (CBP)] depends upon phosphorylation of CREB at ser133 (Chrivia et al., 1993; Arias et al., 1994; Kwok et al., 1994). The extent of CREB binding to CREs is regulated in a tissue- and gene-specific manner and epigenetic mechanisms may play a role in regulating CREB binding to CREs under different circumstances (Cha-Molstad et al., 2004). CREB, and in particular CREB-dependent gene transcription, is thought to be vital for long-term memory (Bourtchuladze et al., 1994).

By 15 min after a stressful challenge such as forced swimming (FS), CREB is phosphorylated extensively in neurons of the dentate gyrus (DG) in the rat hippocampus (Bilang-Bleuel et al., 2000, 2002). Incongruously, FS induced IEGs, c-Fos and Egr-1 are restricted to sparse neurons of the hippocampus (Chandramohan et al., 2008; Gutierrez-Mecinas et al., 2011). It is presently unclear why, if CREB is phosphorylated/activated in virtually all DG granule neurons and is essential for the induction of IEGs, expression of c-Fos and Egr-1 would only occur in sparse DG neurons. Possibly, there are other mechanisms controlling accessibility of pCREB to the genome and/or its transactivation potential in specific neurons. Given that chromatin conformation changes are known to be associated with *c-Fos* induction (Chen and Allfrey, 1987; Clayton et al., 2000), epigenetic regulation of chromatin could be an important factor. Epigenetic regulation could be controlling in which neurons pCREB is allowed to access *c-Fos* (and *Egr-1*) gene CREs in conjunction with required co-factors, thereby allowing transactivation and gene transcription (Reul et al., 2009).

Early work *in vitro* has shown that the induction of *c-Fos* and *Egr-1* requires the formation of phosphorylation and acetylation marks within histone H3 in their gene promoter regions (Mahadevan et al., 1991; Thomson et al., 1999). This dual histone mark is histone H3 phosphorylated at serine10 (S10) and acetylated at lysine14 (K14), i.e., H3K14ac-S10p. Several years ago, we observed in a series of studies *in vivo*, that stressful challenges like novelty and FS resulted in both H3S10p-K14ac formation and c-Fos/Egr-1 induction in the same sparsely distributed granule neurons of the DG (Bilang-Bleuel et al., 2005; Chandramohan et al., 2007, 2008; Gutierrez-Mecinas et al., 2011). Some of this work was repeated in the Morris Water Maze (MWM) using an antibody against a similar mark, namely H3K9ac-S10p, due to the original antibody (H3S10p-K14ac) losing specificity (Carter et al., 2015). These studies showed a similar sparse staining pattern of H3K9ac-S10p and IEG proteins occurring after MWM to that previously observed after FS (Bilang-Bleuel et al., 2005; Chandramohan et al., 2007; Chandramohan et al., 2008; Gutierrez-Mecinas et al., 2011; Carter et al., 2015). Together these studies and others indicate that the induction of these IEGs is involved in the consolidation of memories in both the FS and MWM tests (Jones et al., 2001; Chandramohan et al., 2008; Reul et al., 2009; Gutierrez-Mecinas et al., 2011; Reul, 2014). The formation of the dual histone mark and IEG protein induction in these neurons after FS was shown to depend on mitogen- and stress-activated kinase 1/2 (MSK1/2) and Elk-1 phosphorylation, which was brought about by concurrent activation of the ERK MAPK and the glucocorticoid receptor pathways (Bilang-Bleuel et al., 2005; Chandramohan et al., 2007, 2008; Gutierrez-Mecinas et al., 2011). Therefore, the dual phospho-acetyl modification of histone H3 has been linked to the initiation of transcription of IEGs, however, how exactly the H3K9/K14ac-S10p modification promotes transcription of IEGs is as yet unknown. Phosphorylation and acetylation of histone H3 leads to a reduction in electrostatic charges, which may attenuate interactions between histone core molecules and the DNA, thereby facilitating chromatin remodeling and transcription factor binding to IEG promoters (Reul et al., 2009). The ability of the chromatin to be modified in this way could exert a layer of control over which areas of DNA can be accessed under different conditions, and subsequently, over which genes have potential for being expressed under different conditions.

This study aimed to investigate, using chromatin immuno-precipitation (ChIP), the hypothesis that formation of phospho-acetylated histone H3 after FS occurs in proximity to CREs within IEG promoters, opening the sites up for pCREB binding, CBP recruitment and transcriptional activation only in the sparse, epigenetically primed neurons. We investigated whether this mark is specific for the CRE region of IEG gene promoters and whether the dual histone mark correlates with increased transcription factor and co-factor binding at these sites. As SRF, in complex with pElk1, can also be involved in initiating IEG transcription (Latinkic et al., 1996; Ling et al., 1998; O’Donnell et al., 2008), we also investigated phospho-acetylated histone H3 formation and pElk1 binding at SREs in IEG promoters.

## Materials and Methods

### Animals

Male Wistar rats (150–225 g on arrival) were purchased from Harlan (Oxon, United Kingdom) and group-housed (2–3 per cage). Rats were housed under standard lighting (lights on 05:00–19:00 h, approximately 100 Lux) and environmentally controlled conditions (temperature 21 ± 1°C; relative humidity 40–60%) with food and water available *ad libitum*. All procedures were approved by the University of Bristol Ethical Committee and by the Home Office of the United Kingdom (UK Animal Scientific Procedures Act, 1986). All rats were handled for at least 4 days prior to experimentation in order to reduce non-specific handling stress on the day of the experiment.

### Forced Swimming

Experiment rats were forced to swim for 15 min in individual glass beakers (height 35 cm, diameter 21.7 cm) filled with water (25°C ± 1) to a depth of ∼21 cm. Subsequently, rats were removed from the beakers, gently towel-dried and returned to their home cages. Baseline groups of rats were left undisturbed in their home cages in the holding room during this time. Rats were killed either at baseline, or at various times after the start of FS (see figure legends for time points). Rats were killed by decapitation following quick (<15 s) anesthesia with isoflurane.

### Tissue Preparation

For RNA analysis, the brain was removed from the skull and cut into 1 mm coronal slices using a brain matrix. The dorsal DG, the dorsal cornu ammonis (CA regions) and the ventral hippocampal region were then micro-dissected from these slices on a stainless steel box filled with ice using Dumont #7 forceps (Fine Science Tools, Vancouver, BC, Canada) under a dissection microscope (Leica M29s, Leica Biosystems). DG and CA tissue was snap frozen in tubes in liquid nitrogen and then stored at -80°C. The region micro-dissected spanned the coordinates (anterior-posterior) -2.92 and -3.96 mm from Bregma, according to the atlas of Paxinos and Watson (Paxinos and Watson, 2007).

For ChIP analysis, the brain was removed from the skull and the whole hippocampus was dissected and rapidly snap-frozen in liquid nitrogen before being transferred to -80°C for storage.

### RNA Analysis

Unless otherwise stated, all reagents for this study were purchased from Sigma–Aldrich (Dorset, United Kingdom). RNA extraction and analysis was carried out as described previously (see ref Carter et al., 2015) using the TRI reagent method as per manufacturer’s instructions. Briefly, dorsal DG and CA region tissue samples were homogenized in TRI reagent (using 1 ml for CA regions and 500 μl for DG), 1-bromo-3-chloropropane was used for separation of the RNA containing phase and 2-propanol was used to precipitate the RNA. RNA pellets isolated using this method were air-dried and then dissolved in 30 μl of nuclease-free water (Life Technologies, Paisley, United Kingdom). Samples were analyzed for RNA integrity with an Agilent 2100 Bioanalyser (Agilent Technologies, Cheshire, United Kingdom); all RNA samples analyzed in this way had RNA integrity numbers (RIN) of between 8.1 and 8.9. RNA concentrations were measured using a NanoPhotometer Pearl (Implen, Munich, Germany). One microgram of RNA was converted to cDNA using a QuantiTect reverse transcription kit (Qiagen, Manchester, United Kingdom) according to manufacturer’s instructions. This conversion also included a step to remove any DNA contamination before the reversal.

Quantitative polymerase chain reaction (qPCR) was carried out in a StepOnePlus real-time PCR machine (Life Technologies). In each well of a 96 well plate, 2 μl of cDNA was added to 18 μl of reaction solution [1x TaqMan fast advanced mastermix (Life Technologies), 900 nM forward and reverse primers, 250 nM dual labeled probe (FAM/TAMRA) in nuclease free water]. Primers and probes were designed using Primer Express software (version 3.0, Life Technologies). Tyrosine 3-Monooxygenase/Tryptophan 5-Monooxygenase Activation Protein Zeta (*Ywhaz*) and hypoxanthine phosphoribosyltransferase 1 (*Hprt1*) were used as reference genes. See **Table 1** for cDNA primer and probe sequences. Relative mRNA expression was calculated using the Pfaffl method (Pfaffl, 2001), normalizing the PCR readouts for the target genes to those of the reference genes.

**Table 1:** Primers and probe sequences used in RNA analysis, including gene symbol and accession number for the target genes.

Gene symbol	Accession number	Forward primer sequence	Reverse primer sequence	Probe sequence
Ywhaz	NM_013011.3	TGCTGCTGGTGATGACAAGAA	CATCTCCTTTTTGCTGATTTCAAA	TGGACCAGTCACAGCAAGCATACCAAGAA
Hprt1	NM_012583.2	CCTCCTCAGACCGCTTTTCC	CATAACCTGGTTCATCATCACTAATCA	CATGTCGACCCTCAGTCCCAGCG
*c*-Fos	NM_022197.2	CAGCCAAGTGCCGGAATC	GCAACGCAGACTTCTCGTCTT	ATACGCTCCAAGCGGAGACAGATCAACTT
Egr-1	NM_012551.2	AAGACACCCCCCCATGAAC	CTCATCCGAGCGAGAAAAGC	CCCGTATGCTTGCCCTGTTGAGTCC

### Chromatin Immunoprecipitation

ChIP was essentially conducted as described previously (Carter et al., 2015; Mifsud and Reul, 2016). For each preparation of chromatin, the hippocampi of two rats were cross-linked for 10 min in a 1% formaldehyde buffer (containing 5 mM sodium butyrate (NaBut), 0.1 mM PMSF, PhosStop phosphatase inhibitor cocktail [1 tablet/10 ml, Roche) in 1x phosphate-buffered saline (PBS)]. Cross-linking of the tissue was stopped by adding glycine to a final concentration of 200 mM. Samples were then centrifuged for 5 min at 7500 rpm at 4°C and the resulting pellets were washed three times with ice cold PBS solution [containing 5 mM NaBut, 1 mM NaV_3_O_4_, 0.1 mM PMSF, PhosStop phosphatase inhibitor cocktail (1 tablet/10 ml)]. The pellet was then re-suspended in 1 ml of ice cold lysis buffer [containing 50 mM Tris-HCl pH 8.0, 150 mM NaCl, 5 mM EDTA, 0.5% Igepal, 0.5% sodium deoxycholate, 1% SDS, 5 mM NaBut, 2 mM AEBSF, 1 mM NaV3O4, 1x dilution of protease inhibitor solution from Complete Ultra EDTA-free protease inhibitor tablets (Roche), PhosStop phosphatase inhibitor cocktail (1 tablet/10 ml)] and incubated for 15 min at 4°C.

Each sample was then separated into 200 μl aliquots and sonicated for 3 × 10 cycles (30 s on, 60 s off, on high power). Tubes were gently vortexed in between sets of cycles. After sonication, the recombined samples were centrifuged for 10 min at 14,000 rpm, 4°C and the supernatant (the chromatin) was collected, aliquoted, and snap frozen in dry ice and stored at -80°C.

For ChIP, a 200 μl aliquot of chromatin was combined with 1800 μl of dilution buffer [containing 50 mM Tris-HCl pH 7.5, 150 mM NaCl, 5 mM EDTA pH 7.5, 1% triton, 0.1% sodium deoxycholate, 5 mM NaBut, 1 mM AEBSF, 1x dilution of protease inhibitor solution from Complete Ultra EDTA-free protease inhibitor tablets (Roche), PhosStop phosphatase inhibitor cocktail (1 tablet/10 ml)] and 10 μl of antibody (Histone H3 acetyl K9 phospho S10 (Abcam), pCREB rabbit polyclonal (Millipore), CBP rabbit polyclonal [(A22) X, Santa Cruz], CREB-1 rabbit polyclonal [(C-21) X Santa Cruz], pElk-1 mouse monoclonal [(B-4) X; Santa Cruz)], the samples were then rotated overnight at 4°C. Each of these antibodies are ChIP grade and have been used successfully for ChIP in the past (Zhang et al., 2008; Arampatzi et al., 2013; Kabir et al., 2013; Zimmermann et al., 2015). Immunohistochemical tests have shown that the anti-H3K9ac-S10p antibody produces the same sparse DG staining pattern as that published for anti-H3S10p-K14ac (Carter et al., unpublished observations) (Chandramohan et al., 2008; Gutierrez-Mecinas et al., 2011; Carter et al., 2015). In separate tubes (one for each chromatin sample), 150 μl of Protein A magnetic Dynabeads (Life Technologies) - except in the case of pElk-1, where Protein G Dynabeads (more specific for the mouse antibody, Invitrogen) were used - were washed once in 0.5% BSA in PBS and then left to rotate overnight in 0.5% BSA in PBS at 4°C.

The following day, the beads were washed with ice-cold dilution buffer (containing 50 mM Tris-HCl pH 7.5, 150 mM NaCl, 5 mM EDTA pH 7.5, 1% triton, 0.1% sodium deoxycholate) and the chromatin/ antibody mixture was added to the beads and rotated at 4°C for 3 h. The beads were then collected using a magnetic stand and the unbound liquid was removed. The beads were washed three times with RIPA wash buffer [containing 10 mM tris pH 7.5, 1 mM EDTA pH 7.5, 0.1% SDS, 0.5 mM EGTA, 1% triton, 0.1% sodium deoxycholate, 140 mM NaCl, 1 mM AEBSF, 1x dilution of protease inhibitor solution from Complete Ultra EDTA-free protease inhibitor tablets (Roche), PhosStop phosphatase inhibitor cocktail (1 tablet/10 ml)] and twice with 1x Tris-EDTA buffer. The chromatin was then eluted from the beads using 200 μl of elution buffer 1 (containing 10 mM tris pH 7.5, 50 mM NaCl, 1.5% SDS) and 100 μl of elution buffer 2 (containing 10 mM Tris, 50 mM NaCl, 0.5% SDS) sequentially, each with 15 min of incubation at room temperature (RT) with regular vortexing. Bound fractions released with each elution buffer were pooled and the crosslinks were reversed by adding NaCl to a final concentration of 200 mM and heating at 65°C overnight. The bound samples were then treated with RNase A (60 μg/ml, 1 h at 37°C) and proteinase K (250 μg/ml, 3 h at 37°C).

Input DNA samples were prepared from 20 μl of chromatin, diluted in 280 μl of 1x Tris EDTA buffer (TE) after which NaCl was added to a final concentration of 200 mM and input samples were incubated overnight at 65°C to reverse the DNA-protein crosslinks. The Input samples were then incubated with RNase A (60 μg/ml, 1 h at 37°C) and proteinase K (250 μg/ml, overnight at 37°C).

Input and Bound samples were purified using a QIAquick PCR purification kit (Qiagen, Manchester, United Kingdom) according to the manufacturer’s instructions. DNA from both Input and Bound samples was quantified using a high-sensitivity double-stranded DNA assay kit using a Qubit 2.0 fluorometer (Life Technologies). Bound DNA products and inputs were diluted to 0.05 ng/μl and 0.5 ng/μl respectively in nuclease-free water (nfH_2_O).

Levels of target gene DNA within Bound and Input fractions was quantified by qPCR using primers and dual labeled probes modified with FAM as the fluorescent dye and TAMRA as the quencher. Primers and probes were designed using Primer Express software (version 3.0), see **Table 2** for primer and probe sequences used after ChIP. Primers were designed to cover CRE and serum responsive element (SRE) sites within the IEG promoters. qPCR was carried out as described for RNA analysis, however, a standard curve was also run, using a serial dilution of rat brain genomic DNA (BioChain, CA, United States). Enrichment was calculated using the following equation: Bound fraction/ Input fraction (B/I).

**Table 2:** Primers and probe sequences used in ChIP analysis, including gene symbol and Ensembl gene ID for the target genes.

Gene symbol	Ensembl gene ID	Forward primer sequence	Reverse primer sequence	Probe sequence
*c*-Fos SRE	ENSRNOG00000008015	TCAATCCCTCCCTCCTTTACAC	GGTTCCCCCCAGGACAAC	AGGTTTCCACGGCCGGTCCC
*c*-Fos CRE	ENSRNOG00000008015	TTCCCCCCTCCAGTTTCTCT	TCAGCTGGCCGCTTTATAGAAG	TTCCGCTCATGACGTAGTAAGCCATTCAA
Egr-1 SRE only	ENSRNOG00000019422	GACCCGGAAACACCATATAAGG	AAGGCGCTGCCCAAATAAG	AAGGATCCCCCGCCGGAACAG
Egr-1 CRE/SRE	ENSRNOG00000019422	GCTCTTGGATGGGAGGTCTTC	TCCGCCGTGACGTACATG	TCCTCCCGGTCGGTCCT
BDNF CRE^∗^	ENSRNOG00000047466	TCATGACACCAGACAAATCCTGT	TCTCCCTAGAGGCAGTGTGACA	TTCCCTATCTGAAAAACACCGTGGTCCG

*^∗^CRE site (TGACGGC) predicted by Genomatix software located near the transcriptional start site of exon IX in BDNF.*

### Statistical Analysis

The statistical and graphical package used to analyze data was GraphPad Prism 6 (GraphPad Software, San Diego, CA, United States). Relative RNA expression is presented as a fold-change over baseline levels, after normalization to reference genes, *YWHAZ* and *Hprt1*. ChIP data is expressed as fold change in enrichment over baseline levels. Graphs present group means ± SEM, sample sizes and individual statistical results are stated within the figure legends. All data were analyzed using One-way ANOVA followed by Dunnet’s or Bonferroni *post hoc* tests, or by unpaired Student’s *t*-tests as appropriate. In all cases *p* < 0.05 was considered to be statistically significant.

## Results

### Forced Swimming Induces IEG mRNA Expression in Hippocampal Sub-regions

**FIGURE 1:**
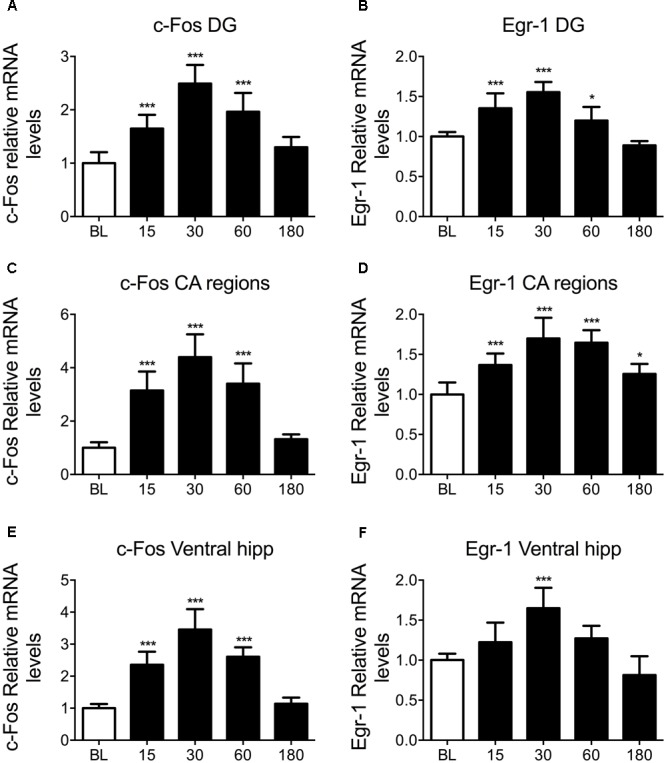
Changes in *c-Fos* and *Egr-1* mRNA in the hippocampus in response to FC. Rats were killed either under baseline conditions, or 15, 30, 60, or 180 min after the start of FC (15 min in 25°C water). The graphs show mean fold-change over BL mRNA levels (mean ± SEM, *n* = 7–9) of **(A)** *c-Fos* in the DG of the dorsal hippocampus, **(B)** *Egr-1* in the DG of the dorsal hippocampus, **(C)** *c-Fos* in the CA regions of the dorsal hippocampus, **(D)** *Egr-1* in the CA regions of the dorsal hippocampus, **(E)** *c-Fos* in the ventral hippocampus and **(F)** *Egr-1* in the ventral hippocampus. Relative mRNA expression over housekeeping genes (HPRT1 and YWHAZ) was calculated following qPCR using the Pfaffl method. Statistical analysis: One-way ANOVA; **(A)** *F*_(4,38)_ = 36.47, *p* < 0.0001, **(B)** *F*_(4,38)_ = 36.58, *p* < 0.0001, **(C)** *F*_(4,39)_ = 46.87, *p* < 0.0001, **(D)** *F*_(4,38)_ = 22.80, *p* < 0.0001, **(E)** *F*_(4,36)_ = 56.94, *p* < 0.0001, **(F)** *F*_(4,36)_ = 18.77, *p* < 0.0001. Dunnett’s *post hoc* test, ^∗^*p* < 0.05 significantly different from BL, ^∗∗∗^*p* < 0.001 significantly different from BL.

To study IEG mRNA changes in the hippocampus following acute stress, rats were killed at baseline, 15, 30, 60, and 180 min after exposure to 15 min of FS. *c-Fos* and *Egr-1* mRNA changes within the DG of the dorsal hippocampus, the CA of the dorsal hippocampus, and the ventral hippocampus were analyzed. Exposure of rats to FS stress caused a highly significant increase in circulating corticosterone levels which peaked in rats killed 30 min after start of the stressor and returned to baseline levels at 180 min after stress. The plasma corticosterone profile from these animals, including relevant methological details, has been published previously (Mifsud and Reul, 2016). Furthermore, our microdialysis studies *in vivo* have shown that FC results in increased local concentrations of free corticosterone in the rat hippocampus (Droste et al., 2008, 2009, Qian et al., 2011). These observations confirm that 15 min of FS stress was sufficient to induce an acute stress response. In the present study, we found that FC led to significant, transient increases in *c-Fos* and *Egr-1* mRNA in all hippocampal sub-regions studied (**Figure 1**). This was unexpected given the previously reported stress-induced increase in pCREB was located predominantly to neurons in the DG (Bilang-Bleuel et al., 2002). The pattern of mRNA changes was very similar for *c-Fos* and *Egr-1*, showing a rapid mRNA activation profile characteristic of IEGs, peaking 30 min after the onset of stress. Housekeeping gene expression remained stable in all hippocampal regions over all time points (**Supplementary Figure S1**). Due to the similarity in results from different hippocampal sub-regions, whole hippocampal samples were processed in subsequent ChIP experiments. We adopted a similar approach in a recent study (Mifsud and Reul, 2016).

### Forced Swimming Increases H3K9ac-S10p Formation at CRE Sites within IEG Promoter Regions

**FIGURE 2:**
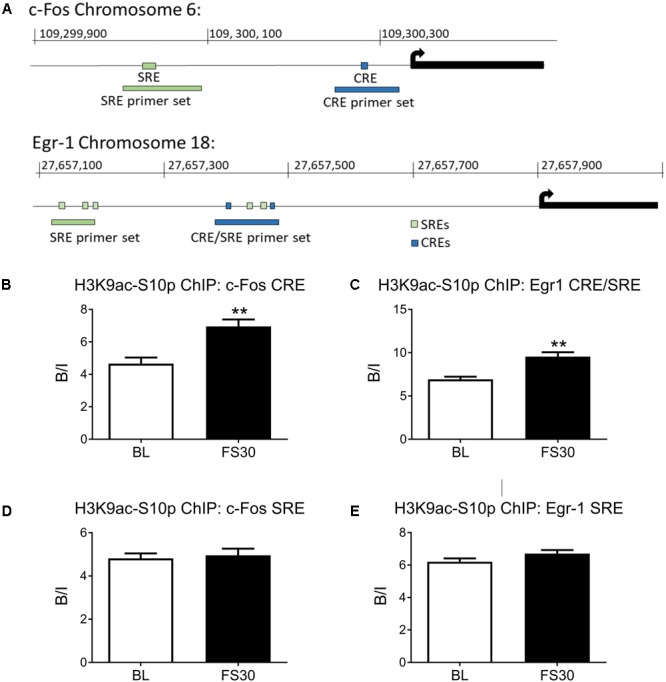
Histone H3 phospho-acetylation in the promoter regions of *c-Fos* and *Egr-1*. Rats were killed either under baseline conditions or 30 min after the start of FC (15 min in 25°C water). Chromatin immuno-precipitation was carried out on whole hippocampal tissue, using an antibody against H3K9ac-S10p. Primers for qPCR were designed to cover Serum Responsive Elements (SREs) and cAMP Responsive Elements (CREs) within the *c-Fos* and *Egr-1* promoters. The location of the SREs, the CREs and the primers in the promoter regions of *c-Fos* and *Egr-1* are shown in **(A)**. The graphs show enrichment of H3K9ac-S10p at **(B)** the *c-Fos* promoter CRE, **(C)** the *Egr-1* promoter region containing both CREs and SREs, **(D)** the *c-Fos* promoter SRE, **(E)** the *Egr-1* promoter region containing SREs. The level of enrichment for each group was calculated by dividing the quantity of target DNA in the H3K9ac-S10p-bound fraction by the quantity of DNA in the input fraction (ratio bound over input, B/I, mean ± SEM, *n* = 3). Statistical analysis: Student’s unpaired *t*-test; **(B)** *t* = 4.04, *p* = 0.0068, **(C)** *t* = 4.52, *p* = 0.004, **(D)** *t* = 0.37, *p* = 0.723, **(E)** *t* = 1.66, *p* = 0.148. ^∗∗^*p* < 0.01 significantly different from BL.

The rat *c-Fos* promoter contains a proximal CRE and a more distal SRE, whilst the *Egr-1* promoter contains two proximal CREs, two proximal SREs and three more distal SREs (**Figure 2A**). Two sets of primers were designed for the *c-Fos* promoter, one set to cover the SRE and one to cover the CRE. Two sets of primers were also designed for the *Egr-1* promoter region, one covering the cluster of SREs and CREs close to the transcriptional start site (named *Egr-1* CRE/SRE primers) and one to cover the more distal SREs (named *Egr-1* SRE only primers).

FC enhances phospho-acetylation of histone H3 in sparse neurons of the hippocampus and is associated with IEG promoter regions (Chandramohan et al., 2008; Reul et al., 2009; Gutierrez-Mecinas et al., 2011). ChIP was performed to investigate whether H3K9ac-S10p formation is occurring in close proximity to SREs or CREs within *c-Fos* and *Egr-1* promoters in hippocampal chromatin after stress. Under baseline conditions there was significant enrichment above input of H3K9ac-S10p [i.e., Bound/Input (B/I) > >1] at both the CRE and SRE sites in both the *cFos* and *Egr1* gene promoters. FS increased the formation of H3K9ac-S10p at the CRE site in the proximal promoter region of *c-Fos*, whilst no stress-induced change in enrichment was seen at the more distal SRE site (**Figures 2B,D**). Similarly, FS also induced an increase in enrichment of H3K9ac-S10p at the region containing CREs and SREs in the *Egr-1* promoter (*Egr-1* CRE/SRE primers), whilst no change in enrichment was seen at the more distal SRE only site (**Figures 2C,E**). A control IgG ChIP was carried out on samples of the same chromatin preparations and no enrichment was found at the CRE sites in either the *c-Fos* or *Egr1* promoter region (B/I ≈1, **Supplementary Figure S2**).

### Forced Swimming Does Not Enhance Recruitment of CREB and CBP to CREs within IEG Promoters

**FIGURE 3:**
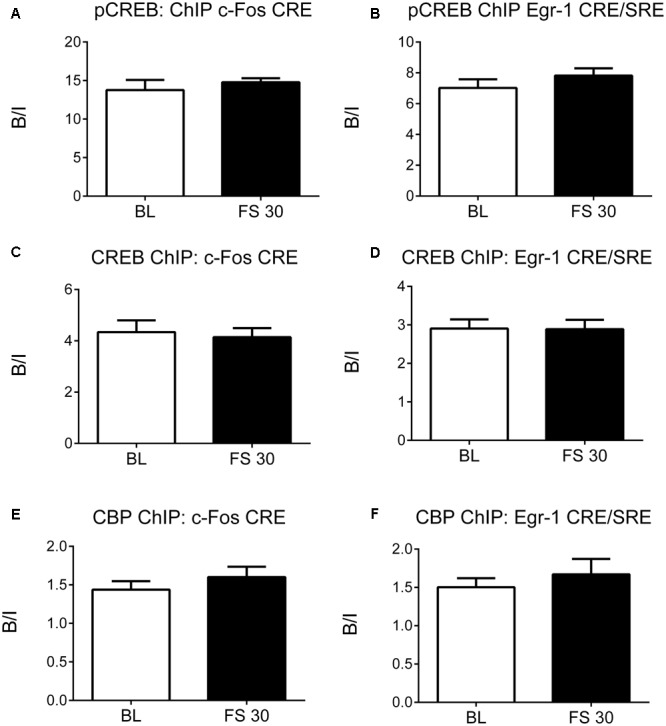
pCREB, CREB, and CBP binding to the CREs in the promoter regions of *c-Fos* and *Egr-1*. Rats were killed either under baseline conditions or 30 min after the start of FC (15 min in 25°C water). Chromatin immuno-precipitation was carried out on whole hippocampal tissue, using antibodies against pCREB, CREB and CBP. Primers for qPCR were designed to cover the CREs in the promoter regions of *c-Fos* and *Egr-1*. The graphs show the enrichment of **(A)** pCREB at the *c-Fos* promoter CRE, **(B)** pCREB at the *Egr-1* promoter CRE/SRE, **(C)** CREB at the *c-Fos* promoter CRE, **(D)** CREB at the *Egr-1* promoter CRE/SRE, **(E)** CBP at the *c-Fos* promoter CRE, **(F)** CBP at the *Egr-1* promoter CRE/SRE. The enrichment for each group was calculated by dividing the quantity of target DNA in the bound fraction by the quantity of DNA in the input fraction (ratio of bound over input, B/I) [mean ± SEM, *n* = 3–6 (*n* = 6 in BL group)]. Statistical analysis: Student’s unpaired *t*-test; **(A)** *t* = 0.78, *p* = 0.4608, **(B)** *t* = 1.10, *p* = 0.3068, **(C)** *t* = 0.34, *p* = 0.7451, **(D)** *t* = 0.04, *p* = 0.9666, **(E)** *t* = 0.95, *p* = 0.3878, **(F)** *t* = 0.77, *p =* 0.4771.

As FS-induced H3K9ac-S10p formation was found to be associated primarily with CREs rather than SREs in the *c-Fos* and *Egr-1* promoters, ChIP studies were carried out to determine the effect of stress on recruitment of CREB and CBP to the CREs in promoter regions of *c-Fos* and *Egr-1* in the hippocampus. Surprisingly, FS did not increase binding of CREB, pCREB, or CBP at the CREs in the *c-Fos* and *Egr-1* promoters at 30 min after stress (**Figure 3**). Apparently, FS did not raise the level of binding beyond the high level of binding displayed by pCREB and CREB already under baseline conditions. In addition, we investigated pCREB binding at *c-Fos* and *Egr-1* promoters at two earlier time points, 5 and 15 min after stress to rule out any earlier FS-induced changes in binding. As with the 30-min data, there was no change in pCREB binding at CREs in the *c-Fos* or *Egr-1* promoters at these time points after stress (**Supplementary Figure S3**). As a positive control, we investigated pCREB binding within a predicted CRE site located at the transcriptional start site of the brain-derived neurotrophic factor (BDNF) transcript IXa. Under baseline conditions, there was no significant enrichment in pCREB binding to the CRE site in *Bdnf*, however, FS stress caused a significant increase in pCREB binding in this gene (**Supplementary Figure S4**). Given this finding was generated using the same purified DNA samples from the pCREB ChIP, it confirms specificity of the high baseline binding and lack of stress-induced increase in pCREB binding at the promoter regions of *cFos* and *Egr1* genes.

### Forced Swimming Does Not Enhance Recruitment of pElk-1 to SREs within IEG Promoters

**FIGURE 4:**
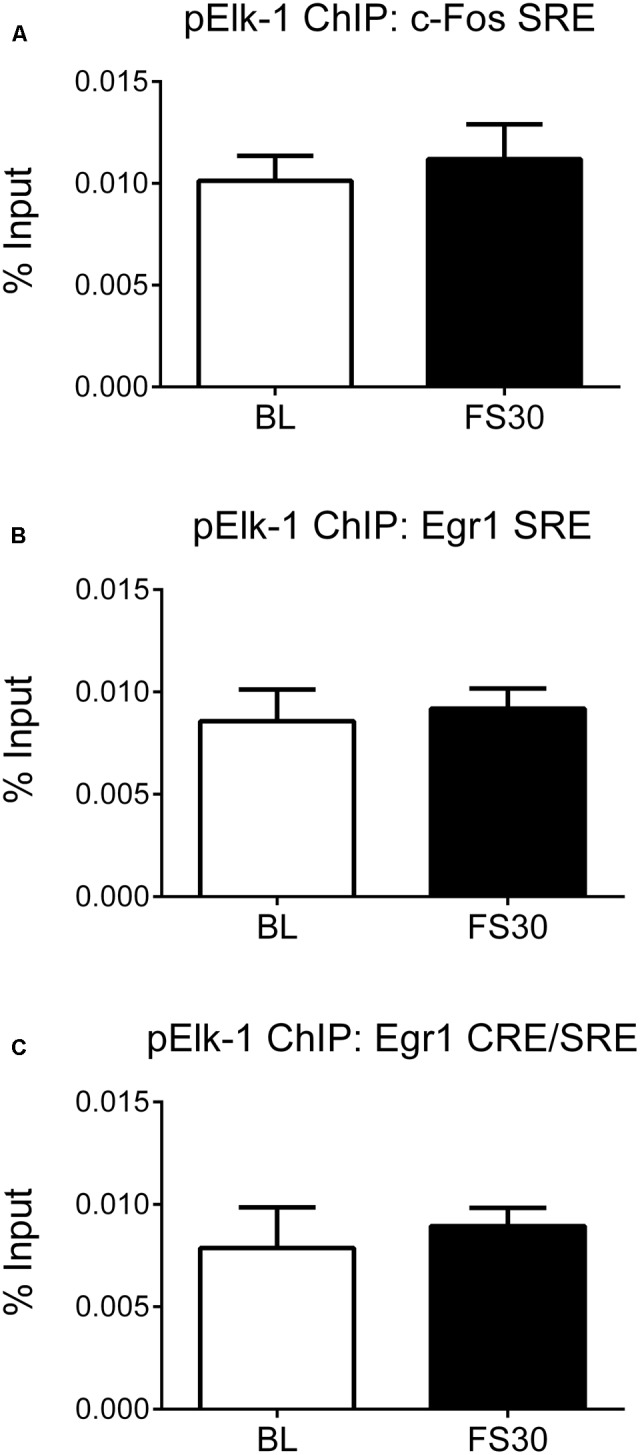
pElk-1 binding at SREs in the promoter regions of *c-Fos* and *Egr-1*. Rats were killed either under baseline conditions or 30 min after the start of FS (15 min in 25°C water). Chromatin immuno-precipitation was carried out on whole hippocampal tissue, using an antibody against pElk-1. Primers for qPCR were designed to cover the SREs in the *c-Fos* and *Egr-1* promoters. In addition, the primers, which cover the CREs in the Erg-1 promoter, also cover two SREs, therefore these primers were also used in this case. Due to low levels of DNA recovered from the ChIP, 2 μl of bound and input were loaded into the PCR mix and the % input method was used to analyze results (see section “Materials and Methods”). The graphs show % input values for pElk-1 at **(A)** the SRE region of the *c-Fos* promoter, **(B)** the distal SRE region of the *Egr-1* promoter, **(C)** the more proximal SRE/CRE region of the *Egr-1* promoter (mean ± SEM, *n* = 3–4). Statistical analysis: Student’s unpaired *t*-test; **(A)** *t* = 0.53, *p* = 0.6219, **(B)** *t* = 0.30, *p* = 0.7768, **(C)** *t* = 0.44, *p* = 0.6797.

It has been shown that SRF, when bound to SRE sites, subsequently recruits pElk-1, a ternary complex factor, to the site (Ling et al., 1998). Furthermore, we have previously shown that FS results in Elk-1 phosphorylation in hippocampal neurons *in vivo* (Gutierrez-Mecinas et al., 2011). Therefore, to explore alternative mechanisms, we investigated whether FS-induced IEG transcription may involve recruitment of pElk to the SRE regions of the promoter. ChIP was performed on hippocampal chromatin prepared from rats killed under baseline conditions and 30 min after FS. There were very low levels of DNA recovered after ChIP, i.e., below detection for dsDNA measurement by the Qubit fluorimetry. Therefore, an alternative method of determining enrichment of target DNA was used and an equal volume of sample of Bound DNA (containing target DNA after ChIP) and Input DNA was used for qPCR followed by calculation of % input (see section “Materials and Methods”). There were, however, no FS-induced changes in pElk-1 binding at the SRE within the *c-Fos* promoter or at either group of SREs within the *Egr-1* promoter (**Figure 4**).

## Discussion

Successful adaptation in response to a stressful event, such as FS in rats, involves molecular changes and *de novo* gene expression within hippocampal neurons. An important step in this process is the induction of the IEGs *c-Fos* and *Egr-1*; genes that are expressed rapidly and sparsely within the hippocampus following a stressor (Gutierrez-Mecinas et al., 2011; Carter et al., 2015). Induction of these genes is under very stringent control involving multiple signaling and epigenetic mechanisms (Gutierrez-Mecinas et al., 2011; Papadopoulos et al., 2011; Carter et al., 2015; Saunderson et al., 2016). Here, we show that FS results in increased phospho-acetylation of histone H3 within the region of CREs in hippocampal *c-Fos* and *Egr-1* promoters; however, this does not appear to lead to increased pCREB or CBP recruitment to those sites. The formation of H3K9ac-S10p occurs specifically within stretches of chromatin encompassing CREs in the IEG promoters, and not at nearby containing SREs, indicating specificity for these CRE only sites. Although previous work has reported a dramatic increase in CREB phosphorylation in the DG at 15 and 30 min after FS (Bilang-Bleuel et al., 2000, 2002; Bachmann et al., 2005), surprisingly this did not manifest into enhanced pCREB binding to the CRE sites within the *c-Fos* and *Egr-1* promoters. The lack of any observed increase in pCREB binding after stress is likely due to the initial high binding of pCREB to these sites under baseline (non-stressed) conditions. Despite the lack of an effect of stress on pCREB binding, we did observe a stress-induced increase in IEG mRNA expression throughout the hippocampus indicating other/additional mechanisms may be controlling the transcription of these genes after stress.

In agreement with our results, another study found no changes in the binding of CREB or pCREB at the *c-Fos* CRE site in frontal cortex, hippocampus and striatum following electroconvulsive shock stress (Tanis et al., 2008). In human glioblastoma T98G cells, however, ChIP in combination with next-generation sequencing showed increased CBP binding to *c-Fos*, after 30 min of stimulation with serum and tetradecanoyl phorbol acetate (TPA) (Ramos et al., 2010). This study unfortunately does not disclose the location within the gene or gene promoter where the increase in CBP binding was found. CREB is thought to be constitutively bound to some CREs within gene promoters, with transactivation enabled after phosphorylation, although there is evidence that phosphorylation can also lead to changes in CREB binding in some cases (Nichols et al., 1992; Hagiwara et al., 1993; Bullock and Habener, 1998; Shaywitz and Greenberg, 1999). *In vivo*, constitutive CREB binding and activity is regulated in a cell- and gene-specific manner, which cannot be predicted on the basis of binding properties observed *in vitro* (Cha-Molstad et al., 2004).

The phosphorylation of CREB at Ser133 has long been thought to be a critical component in activity-dependent IEG expression (Sassone-Corsi et al., 1988; Sheng et al., 1990; Lonze and Ginty, 2002), however, recently doubt has been cast on this assumption. Blendy and colleagues derived a mouse model with a single point mutation that prevents CREB phosphorylation at Ser133 (Ser133Ala mutant mice) (Briand et al., 2015). The mice did not have altered CREB protein levels, or altered cAMP-responsive element modulator (CREM) mRNA levels (as was seen in CREBαΔ mutant mice) (Bourtchuladze et al., 1994; Blendy et al., 1996). Surprisingly, Blendy and colleagues found that the Ser133Ala mutant mice had no deficits in hippocampus- or striatum-dependent fear conditioning learning and in *c-Fos* or *FosB* gene transcription either at baseline or 30 min after fear-conditioning (Briand et al., 2015). These observations indicate that CREB phosphorylation at Ser133 is not of critical importance for stress-related learning and IEG induction. Our findings correspond with this notion.

Our study shows a substantial enrichment of pCREB at CRE sites within the *c-Fos* and *Egr-1* gene promoters under baseline conditions indicating a considerable level of transcription factor binding under these conditions. These observations appear to contradict immunohistochemical results showing very low immuno-reactivity of pCREB at baseline (Bilang-Bleuel et al., 2000, 2002). Presently, it is unknown whether this discrepancy is due to differences in antibody binding and signal detection as a result of inherent differences between the methods used (immuno-precipitation of chromatin versus immuno-detection in tissue sections). Possibly, in tissue sections, under baseline conditions pCREB molecules are located within sections of the chromatin which are not accessible for the antibody. In contrast, for ChIP analysis, the chromatin is sheared and hence pCREB would be accessible in all samples. The high level of pCREB binding to hippocampal chromatin under baseline conditions was unexpected and indicates that these areas of the chromatin are easily accessible to pCREB. The original idea that the H3K9ac-S10p epigenetic modification is acting as a permissive mark for pCREB binding in specific stress-responsive DG neurons, is therefore, improbable. Furthermore, immunohistochemistry presented substantial increases in pCREB immuno-staining in DG neurons after stress (Bilang-Bleuel et al., 2000, 2002; Saunderson et al., 2016), which corresponded with the stress-induced increase in hippocampal IEG transcription we observed but did not correspond with the absence of any change in binding of pCREB at CREs within the two IEG gene promoters. This discrepancy cannot be explained by differences in signal detection and will require further investigation. Given that these IEG CREs are already highly occupied by pCREB under baseline conditions, it is possible that there are hardly any sites available for any additional, stress-induced pCREB molecules to bind. The high level of enrichment of pCREB observed within the promoters of the IEGs under baseline conditions, and the lack of FS-induced increase in pCREB binding at these sites, were not observed in other genes like *Bdnf* indicating that these results are specific for the IEGs studied and are not an artifact of the ChIP procedure itself.

Despite evidence indicating the importance of pCREB in *c-Fos* induction after cellular stimulation *in vitro* (Sheng et al., 1990), increased binding of this transcription factor to CREs within *c-Fos* and *Egr-1* promoters does not appear to be the primary mechanism of stress-induced transcriptional activation of these IEGs in the rat hippocampus *in vivo*. Consequently, the reason for the rise in H3K9ac-S10p formation at these sites after stress remains unclear but does not appear to be a requirement for pCREB binding. Possibly, additional transcription factors and/or other factors (e.g., 14-3-3 proteins) (MacDonald et al., 2005) that interact with phospho-acetylated histone H3 enriched regions may be involved in the transcriptional activation process. Regarding the *c-Fos* gene, such factors may include nuclear factor 1 (NF-1), which has been shown to require histone H3 phospho-acetylation and chromatin remodeling in order to bind to its recognition site within the *c-Fos* gene promoter (O’Donnell et al., 2008, 2012). Additional studies should determine if NF-1 is a critical transcription factor involved in the sparse activation of IEGs after FS *in vivo*.

Due to the rapidity of *c-Fos* gene induction, it is thought that this gene is constantly poised for activation, however, full transcription of the gene is prevented by a strong block to elongation, close to the transcriptional start site (TSS) (Fivaz et al., 2000). Proximal-promoter pausing of RNA polymerase II in the first intron of *c-Fos*, means that the promoter could be constitutively active, with continuous initiation of transcription, albeit with prevention of elongation. Unblocking of RNA polymerase II (or the assembly of an RNA polymerase complex with elongation ability) may therefore be an important regulatory mechanism of stimulus-induced transcription (Pinaud and Mirkovitch, 1998; Fujita and Schlegel, 2010). Release of this RNA polymerase arrest may involve phosphorylation of the RNA polymerase and/or demethylation of cytosine residues within the local DNA sequence (Rountree and Selker, 1997; Irvine et al., 2002; Levine, 2011; Saunderson et al., 2016). In fact, our recent work shows that, after FS, significant DNA demethylation of distinct CpGs occurs in a region where RNA polymerase is paused at the start of transcription of the *c-Fos* gene (Saunderson et al., 2016), providing a possible mechanism for release of the block to elongation. We showed that stress-evoked DNA demethylation may be brought about through recruitment of Dnmt3a (acting as a DNA demethylase) (Metivier et al., 2008; Chen et al., 2013) to the gene promoter and 5′-UTR region of the gene; an epigenetic process that may require local formation of H3K9ac-S10p (Saunderson et al., 2016). Thus, transcriptional elongation, rather than transcriptional initiation, could therefore be a major regulator of *c-Fos* gene transcription and perhaps of other IEGs as well. Pausing of RNA polymerase at the promoter has also been reported for the *Egr-1* gene, with different mechanisms thought to be involved in releasing RNA polymerase to commence transcription in different cell types (Balamotis et al., 2009). *In vivo*, FS was shown to result in recruitment of Dnmt3a and DNA demethylation of the *Egr-1* gene promoter in the hippocampus, which in this case may have been critical for unblocking transcriptional elongation (Saunderson et al., 2016).

In summary, this study has identified that the dual epigenetic mark, H3K9ac-S10p is formed in close proximity to CRE binding sites within *c-Fos* and *Egr-1* gene promoters within the rat hippocampus following FS stress. Furthermore, despite the formation of these marks specifically at CRE sites within these IEG promoters, no increases in CREB, pCREB or CBP binding at these sites were observed after FS stress. Therefore, the original hypothesis that, within the *c-Fos* and *Egr-1* promoters, H3K9ac-S10p is acting as permissive modifications allowing pCREB to bind needs to be rejected. The enhanced formation of H3K9ac-S10p after FS may be involved in the enhanced recruitment of Dnmt3a to the IEG gene promoter/5′-UTR regions. The exact functional significance of H3K9ac-S10p at IEG promoters remains to be determined.

Given the clear stress-induced increase in IEG mRNA expression presented here, together with existing immunohistochemical data for the proteins associated with these genes we propose that constitutive binding of pCREB may contribute to maintaining the IEGs in a poised state of activation, which after stress transcends into full transcriptional activation as a result of distinct stress-evoked epigenetic/transcriptional processes at the IEG gene promoters/5′-UTR regions. Further work is required to fully elucidate alternative mechanisms of control, of which proximal-promoter pausing, DNA demethylation events, and/or the involvement of other transcription factors are viable options.

## Author Contributions

SC, KM, and JR designed the study, performed the research, and wrote the manuscript.

## Conflict of Interest Statement

The authors declare that the research was conducted in the absence of any commercial or financial relationships that could be construed as a potential conflict of interest.

## Supplementary Material

FIGURE S1 Houskeeping gene mRNA expression. Groups of rats were killed either under baseline (BL) conditions or at 15, 30, 60, or 180 min after the start of FS (15 min in 25°C water). The graphs show mean fold-change over BL mRNA levels (mean ± SEM, *n* = 7–9) of **(A)** *Hprt1* in the DG of the dorsal hippocampus, **(B)** *Ywhaz* in the DG of the dorsal hippocampus, **(C)** *Hprt1* in the CA regions of the dorsal hippocampus, **(D)** *Ywhaz* in the CA regions of the dorsal hippocampus, **(E)** *Hprt1* in the ventral hippocampus and **(F)** *Ywhaz* in the ventral hippocampus. Statistical analysis: One-way ANOVA; **(A)** *F*_(4,38)_= 1.549, *p* = 0.2077, **(A)** *F*_(4,38)_= 1.351, *p* = 0.2690, **(C)** *F*_(4,39)_= 1.083, *p* = 0.3784, **(D)** *F*_(4,39)_= 0.6720, *p* = 0.6154, **(E)** *F*_(4,40)_= 0.9432, *p* = 0.4491, **(F)** *F*_(4,40)_= 0.6664, *p* = 0.6190.

FIGURE S2 IgG ChIP control. Rats were killed either under baseline conditions or 30 min after the start of FS (15 min in 25°C water). Chromatin immuno-precipitation was carried out on whole hippocampal tissue using rabbit control IgG. Primers for qPCR were designed to cover Serum Responsive Elements (SREs) and cAMP Responsive Elements (CREs) within the *c-Fos* and *Egr-1* promoters. The graphs show enrichment at **(A)** the *c-Fos* promoter CRE, and **(B)** the *Egr-1* promoter region containing both CREs and SREs. The enrichment for each group was calculated by dividing the quantity of target DNA in the bound fraction by the quantity of DNA in the input fraction (ratio bound over input, B/I) [mean ± SEM, *n* = 3]. Statistical analysis: Student’s unpaired *t*-test; **(A)** *t* = 0.442, *p* = 0.682, **(B)** *t* = 0.671, *p* = 0.539.

FIGURE S3 pCREB binding to the CREs in the promoter regions of *c-Fos* and *Egr-1*. Rats were killed either under baseline conditions or 5 or 15 min after the start of FS (25°C water). Chromatin immuno-precipitation was carried out on whole hippocampal tissue using antibodies against pCREB. Primers for qPCR were designed to cover the CREs in the promoter regions of *c-Fos* and *Egr-1* (**Table 2**). The graphs show the enrichment of **(A)** pCREB at the *c-Fos* promoter CRE, and **(B)** pCREB at the *Egr-1* promoter CRE/SRE. The enrichment for each group was calculated by dividing the quantity of target DNA in the bound fraction by the quantity of DNA in the input fraction (ratio bound over input, B/I), as determined by qPCR analyses (see section “Materials and Methods”) [mean ± SEM, *n* = 4]. Statistical analysis: One-way ANOVA; **(A)** *F*_(2,9)_= 0.1388, *p* = 0.8722, **(B)** *F*_(2,9)_= 0.7502, *p* = 0.4996.

FIGURE S4 pCREB binding within the rat hippocampal *Bdnf* gene. Rats were killed either under baseline conditions or 5 or 15 min after the start of FS (25°C water). Chromatin immuno-precipitation was carried out on whole hippocampal tissue using antibodies against pCREB. Primers for qPCR were designed covering a predicted CRE located near the transcriptional start site for exon IXa within the *Bdnf* gene. The graph shows enrichment of pCREB at the *bdnf* CRE site. The enrichment for each group was calculated by dividing the quantity of target DNA in the bound fraction by the quantity of DNA in the input fraction (ratio bound over input, B/I), as determined by qPCR analyses (see section “Materials and Methods”) [mean ± SEM, *n* = 4]. Statistical analysis: One-way ANOVA; *F*_(2,9)_= 8.066, *p* = 0.0098; Bonferroni *post hoc* test: ^∗∗^*p* < 0.01 compared with the BL group.
