# Supplementary figures and images for: Acute Stress Enhances Epigenetic Modifications But Does Not Affect the Constitutive Binding of pCREB to Immediate-Early Gene Promoters in the Rat Hippocampus
Source: Front Mol Neurosci. 2017 Dec 19;10:416. doi: 10.3389/fnmol.2017.00416 (PMC5742222; doi:10.3389/fnmol.2017.00416)

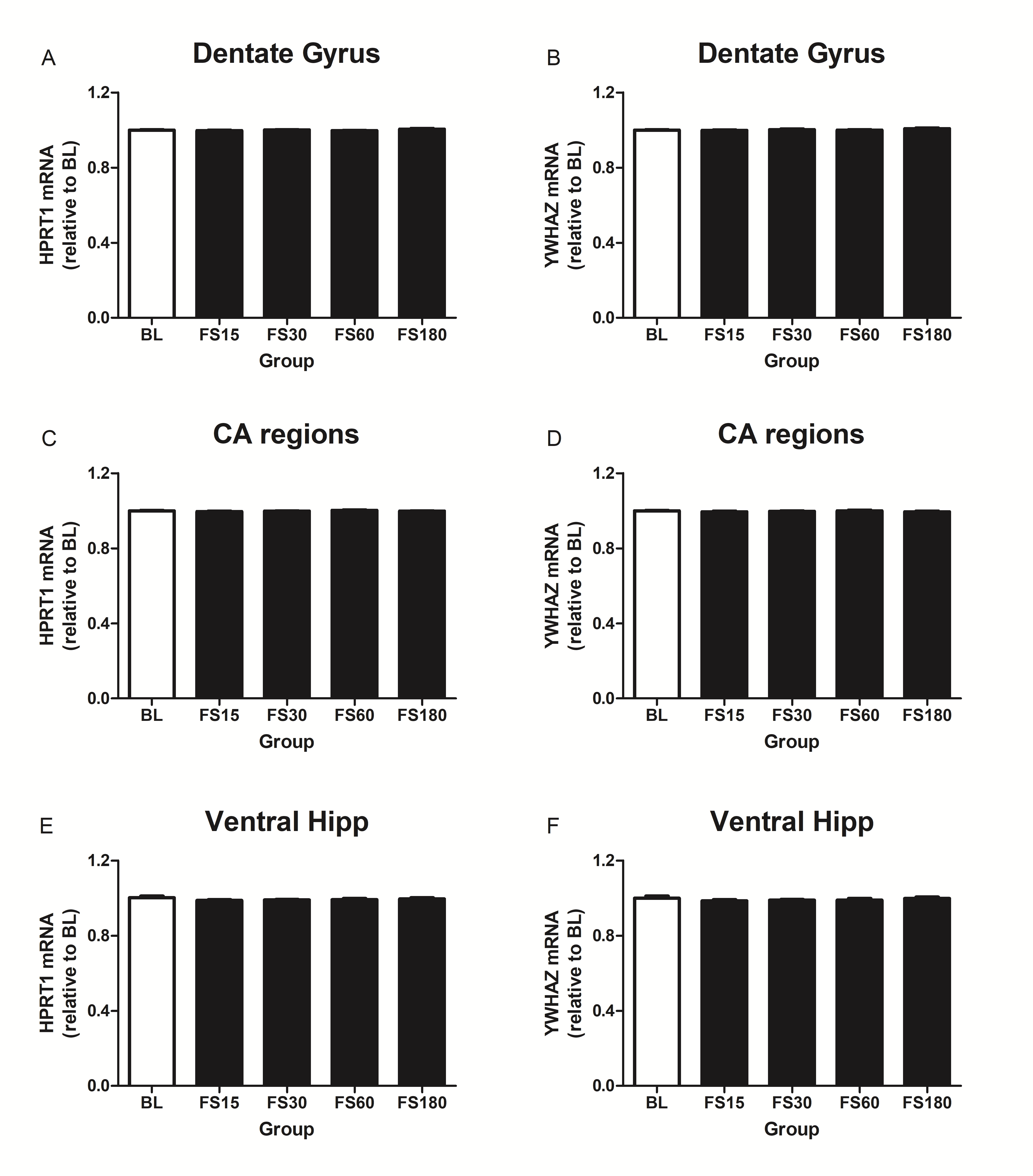

Supplement: FIGURE S1 — Houskeeping gene mRNA expression. Groups of rats were killed either under baseline (BL) conditions or at 15, 30, 60, or 180 min after the start of FS (15 min in 25°C water). The graphs show mean fold-change over BL mRNA levels (mean ± SEM, n = 7–9) of (A) Hprt1 in the DG of the dorsal hippocampus, (B) Ywhaz in the DG of the dorsal hippocampus, (C) Hprt1 in the CA regions of the dorsal hippocampus, (D) Ywhaz in the CA regions of the dorsal hippocampus, (E) Hprt1 in the ventral hippocampus and (F) Ywhaz in the ventral hippocampus. Statistical analysis: One-way ANOVA; (A) F(4,38)= 1.549, p = 0.2077, (A) F(4,38)= 1.351, p = 0.2690, (C) F(4,39)= 1.083, p = 0.3784, (D) F(4,39)= 0.6720, p = 0.6154, (E) F(4,40)= 0.9432, p = 0.4491, (F) F(4,40)= 0.6664, p = 0.6190. [file Image_1.TIF]

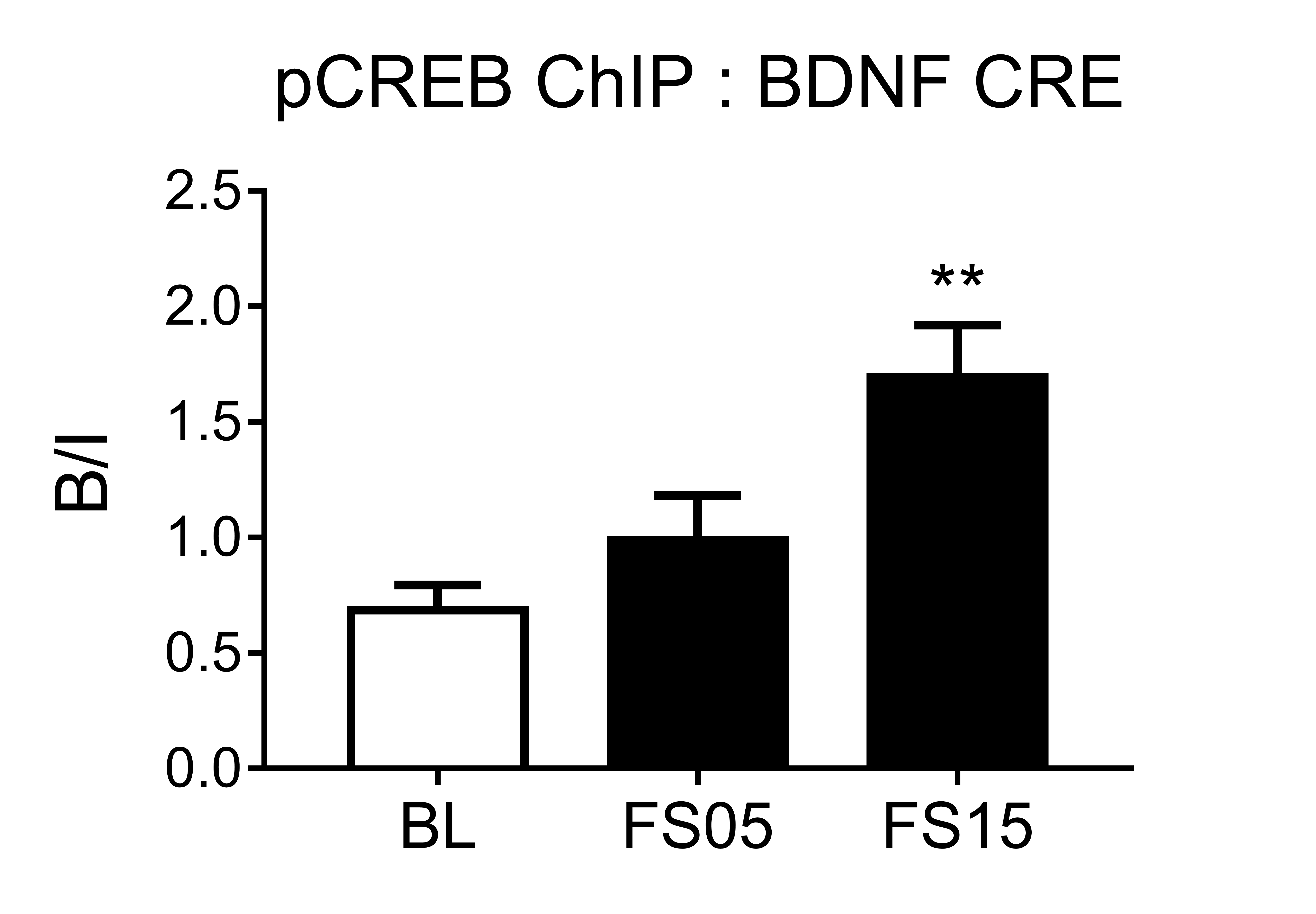

Supplement: FIGURE S4 — pCREB binding within the rat hippocampal Bdnf gene. Rats were killed either under baseline conditions or 5 or 15 min after the start of FS (25°C water). Chromatin immuno-precipitation was carried out on whole hippocampal tissue using antibodies against pCREB. Primers for qPCR were designed covering a predicted CRE located near the transcriptional start site for exon IXa within the Bdnf gene. The graph shows enrichment of pCREB at the bdnf CRE site. The enrichment for each group was calculated by dividing the quantity of target DNA in the bound fraction by the quantity of DNA in the input fraction (ratio bound over input, B/I), as determined by qPCR analyses (see section “Materials and Methods”) [mean ± SEM, n = 4]. Statistical analysis: One-way ANOVA; F(2,9)= 8.066, p = 0.0098; Bonferroni post hoc test: ∗∗p < 0.01 compared with the BL group. [file Image_4.TIF]
